# Complete genome sequences of three commensal and two avian pathogenic *Escherichia coli* strains isolated from farm animals in Russia

**DOI:** 10.1128/MRA.00654-23

**Published:** 2023-10-09

**Authors:** Dmitry Sutormin, Veronika Mihailovskaya, Anna Trofimova, Victor Mamontov, Marina Kuznetsova, Konstantin Severinov

**Affiliations:** 1 Center for Molecular and Cellular Biology, Skolkovo Institute of Science and Technology, Moscow, Russia; 2 Institute of Ecology and Genetics of Microorganisms, Ural Branch of the Russian Academy of Sciences, Perm, Russia; 3 Institute of Gene Biology, Russian Academy of Science, Moscow, Russia; 4 Waksman Institute for Microbiology, Rutgers, the State University of New Jersey Piscataway, New Jersey, USA; 5 Institute of Molecular Genetics, National Research Center "Kurchatov Institute", Moscow, Russia; Queens College, Queens, New York, USA

**Keywords:** *Escherichia coli*, APEC, antibiotic-resistance genes, virulence-associated genes, complete genome

## Abstract

Farm animals are a natural reservoir of commensal and pathogenic *Escherichia coli* strains with high zoonotic potential. Here, we present five complete genomes of *E. coli* strains isolated from healthy animals and animals with colisepticemia from farms in Russia. The strains contain diverse virulence-associated and antibiotic resistance genes and multiple plasmids.

## ANNOUNCEMENT

Farm animals are a reservoir of highly diverse commensal and pathogenic *Escherichia coli*, which are frequently multidrug resistant. *E. coli* antibiotic resistance and virulence-associated genes (ARGs and VAGs, respectively) are often carried by transposable elements and plasmids, pointing to the role of horizontal gene transfer in the emergence of virulent multidrug-resistant strains (1).

Five *E. coli* strains were isolated from animals from industrial and private farms in Russia between 2019 and 2021. Three strains were obtained from fecal samples of healthy birds (Ch1) and cows (C25 and C51), and two strains (APEC20/16 and APEC37) were obtained from internal organs of birds with typical lesions of colisepticemia (2, 3). The research was carried out in accordance with the International Guiding Principles for Biomedical Research Involving Animals.

**Fig 1:**
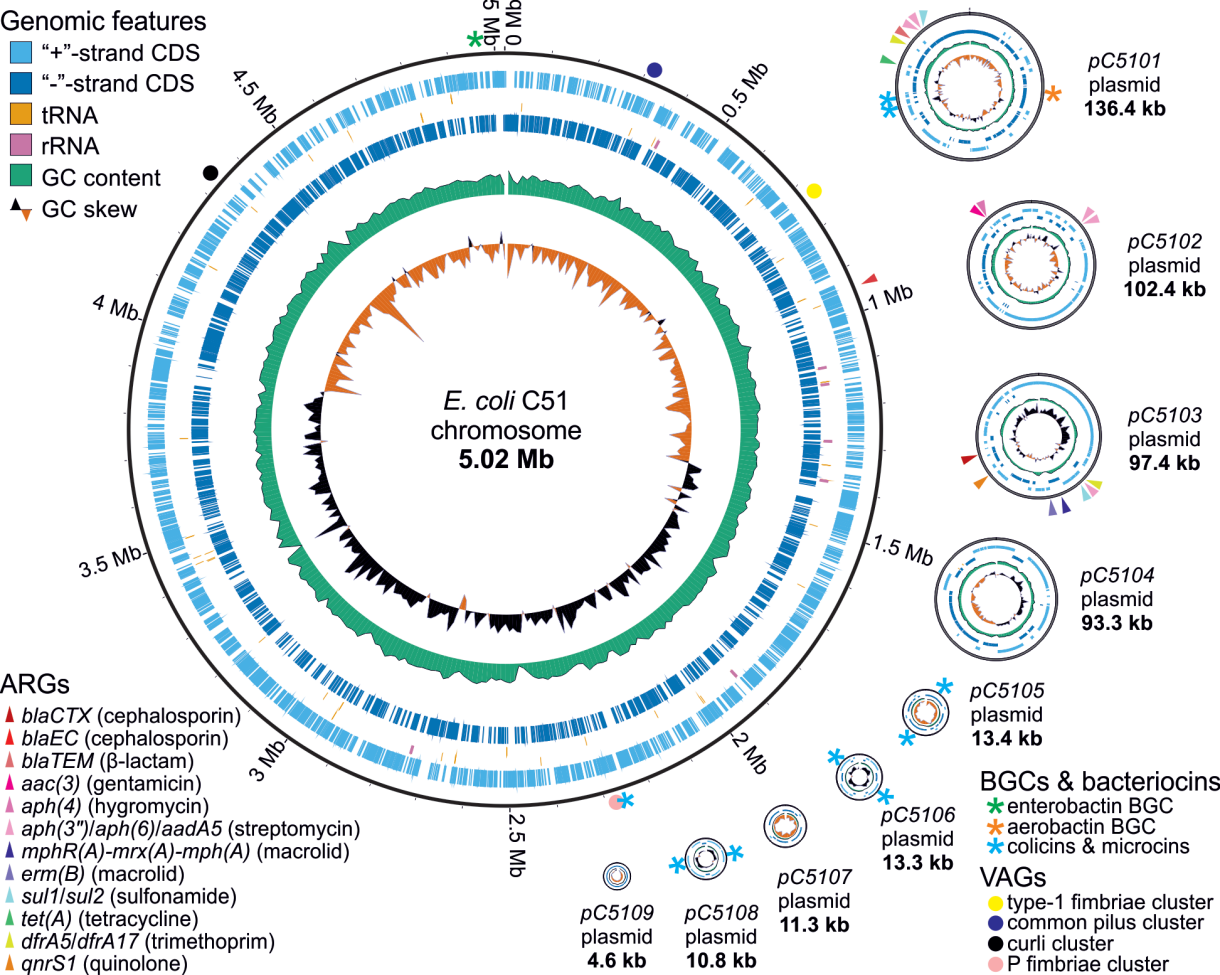
The genome of *E. coli* C51 visualized using GenoVi (7). The chromosomes and plasmids are shown not to scale. Colored triangles, asterisks, and circles indicate the locations of, respectively, ARGs, biosynthetic gene clusters (BGCs), and VAGs. CDS, coding sequence.

Isolation, identification, and cultivation of strains were performed essentially as was described earlier (2, 3). Genomic DNA was extracted from overnight cultures grown at 37°C in lysogeny broth (LB) using GeneJET Genomic DNA Purification Kit (ThermoFisher). Libraries for Nanopore sequencing were prepared directly from extracted DNA using the Native Barcoding Kit (SQK-NBD114-24). Final libraries were enriched for long fragments using the long fragment buffer (LFB). MinION sequencing was performed using R10.4.1 flow cell (FLO-MIN114) with a translocation rate of 400 bps. Base-calling was performed using Guppy v6.0.1 (4) in the “hac” mode. Default parameters were used for all software unless otherwise specified. Draft genomes were assembled with Flye from reads with a quality score >9 (v2.9.1) (5). The assembly was subsequently polished with medaka (v1.7.2), and assembly graphs were inspected in Bandage (v0.8.1) (6). Final assemblies containing circular replicons were annotated using PGAP (v6.1). The sequenced strains contained at least one and up to nine plasmids (Table 1; Fig. 1).

**TABLE 1:** Accession numbers, sequencing and assembly statistics, and genomic features of the sequenced *E. coli* strains^*a*^

	*E. coli* Ch1	*E. coli* C25	*E. coli* C51	APEC20/16	APEC37
**BioProject accession no**.	PRJNA980462				
**BioSample accession no**.	SAMN35637910	SAMN35637911	SAMN35637912	SAMN35637913	SAMN35637914
**SRA accession no**.	SRR24837714	SRR24837713	SRR24837712	SRR24837711	SRR24837710
**GenBank accession no**.	CP127316–CP127320	CP127314–CP127315	CP127304–CP127313	CP127297–CP127300	CP127301–CP127303
**Total reads length (Mb**)	831	987	935	837	816
**No. of reads**	83,046	137,852	107,766	93,032	97,363
**Reads N50 (kb**)	18.2	10.5	13.6	15.6	14.1
**Total chromosome length (bp**)	5,386,706	5,214,475	5,021,543	4,941,568	5,055,174
**GC content (%**)	50.6	50.5	50.8	50.6	50.4
**No. of CDSs (protein**)	5,081	4,653	4,915	4,797	4,826
**No. of tRNAs**	91	87	90	95	88
**No. of rRNAs (5S; 16S; 23S**)	8; 7; 7	7; 7; 7	8; 7; 7	8; 7; 7	8; 7; 7
**No. of CRISPR arrays**	2 (Type I-E)	0	2 (Type I-E)	1 (Type I-E)	2 (Type I-E)
**Plasmids**	4	1	9	3	2
**Plasmid length (bp**)	156,023 72,590 65,488 11,176	29,146	136,371 102,372 97,376 93,338 13,351 13,293 11,304 10,798 4,644	143,113 102,043 93,836	192,726 66,741
**Predicted antibiotic resistance (Abricate**)	Cephalosporin (blaEC-8, chr), sulfonamide (sul2, plasmid), tetracycline [tet(A), plasmid], trimethoprim (dfrA17, plasmid)	Beta-lactam (blaTEM-235, plasmid), cephalosporin (blaEC-5, chr)	Beta-lactam (blaTEM-1, plasmid), cephalosporin (blaEC-18, chr; blaCTX-M-15, plasmids), gentamicin [aac(3)-IVa, plasmid], Hygromycin [aph(4)-Ia, plasmid], macrolide [mph(A), erm(B), plasmid], streptomycin [aph(3'')-Ib, aph(6)-Id, aadA5, plasmids], sulfonamide (sul1, sul2, plasmid), tetracycline [tet(A), plasmid], trimethoprim (dfrA5, dfrA17, plasmids), quinolone (qnrS1, plasmid)	Beta-lactam (blaTEM-1, plasmid), cephalosporin (blaEC-18, chr), fosfomycin (fosA7.5, chr), macrolide [mph(A), plasmid], streptomycin [aadA1, aph(3'')-Ib, aph(6)-Id, plasmid], sulfonamide (sul1, plasmid), tetracycline [tet(A), plasmid], trimethoprim (dfrA1, plasmid)	Cephalosporin (blaEC-15, chr), chloramphenicol (catA1, chr), colistin (mcr-1.1, plasmid), gentamicin [aac(3)-IId, chr], streptomycin (aadA2, chr), sulfonamide (sul1, chr), trimethoprim (dfrA12, chr)
**Predicted VAGs**	*csgDEFG* and *csgBAC* (curli), *ecpRABCDE* (common pilus), *fimB*, *fimE*, *fimAICDFGH* (type 1 fimbriae), *vat* (hemoglobin protease)	*ecpRABCDE* (common pilus), *sfaC*, *sfaB*, *focAICDFGH*, *sfaY* (F1C fimbriae), *papI, papBAHCDJKEFGX* (P fimbriae), *hlyCABD* (hemolysin), *kpsFEDUCS*, *kpsMT* (K1 capsule), *vat* (hemoglobin protease)	*csgDEFG* and *csgBAC* (curli), *ecpRABCDE* (common pilus), *fimB*, *fimE*, *fimAICDFGH* (type 1 fimbriae), *papI, papBAHCDJKEFGX* (P fimbriae)	*csgDEFG* and *csgBAC* (curli), *ecpRABCDE* (common pilus), *fimB*, *fimE*, *fimAICDFGH* (type 1 fimbriae)	*fimB*, *fimE*, *fimAICDFGH* (type 1 fimbriae), *ecpRABCDE* (common pilus)
**Predicted siderophore BGCs**	Aerobactin, enterobactin (chr)	Yersiniabactin, aerobactin, enterobactin (chr)	Enterobactin (chr), aerobactin (plasmid)	Enterobactin (chr)	Enterobactin (chr), aerobactin (plasmid)
**Predicted bacteriocin and microcin BGCs**	MccB17 (plasmid)	Microcin H47, microcin M, colicin E9 (chr), MccC7 (plasmid)	Microcin M (chr), colicin E9, colicin-10, colicin V, colicin Ia, colicin D (plasmids)	Сolicin V, colicin M (plasmid)	Colicin E9 (plasmid)

^*a*^ Chr indicates chromosomal position of a gene or a gene cluster.

Biosynthetic gene clusters (BGCs) and bacteriocins were predicted using antiSMASH (8), PRISM4 (9), and BAGEL4 (10). Several non-ribosomal peptide synthetase and polyketide synthase BGCs for siderophore biosynthesis (yersiniabactin, aerobactin, and enterobactin) and two ribosomally synthesized and post-translationally modified peptide (RiPP) BGCs for microcin B17 and C7 were identified in the sequenced genomes (Table 1). Siderophore BGCs were typically chromosomal, except for the aerobactin BGCs located on plasmids in strains C51 and APEC37. BGCs of RiPPs were predominantly plasmid borne. Six out of 12 identified colicin clusters were found in the C51 strain, which harbors an especially rich set of plasmids (nine plasmids), carrying five out of its colicin clusters (Fig. 1).

Multiple ARGs were predicted with Abricate (11) using the NCBI AMRFinderPlus database (12). Strains C51 and APEC contained a wide range of ARGs which confer resistance to up to eight classes of antibiotics (Table 1) and are frequently organized in resistance islands (Fig. 1). VAGs were predicted using VRprofile2 (13). A diverse repertoire of gene clusters related to cell adhesion (encoding curli, common pilus, type-1, F1C, and P fimbriae) and other genes linked to pathogenicity (hemoglobin protease, hemolysin, and K1 capsule biosynthesis) was detected (Table 1). Interestingly, strains isolated from animals with colisepticemia did not have an increased diversity of VAGs and ARGs compared with isolates from healthy animals, indicating that healthy farm animals can serve as a reservoir of pathogenic and antibiotic-resistant *E. coli*.

## Data Availability

Raw reads obtained in this study were deposited in the NCBI Sequence Read Archive (SRA) under accession numbers SRR24837710-SRR24837714. Annotated genome assemblies were deposited in the NCBI BioProject PRJNA980462.
